# Personality Traits and Their Association With Medication Non‐Adherence in Patients With Multiple Sclerosis

**DOI:** 10.1002/cns.71020

**Published:** 2026-07-10

**Authors:** Pegah Mashhadiakbar, Michael Hecker, Niklas Frahm, Julia Baldt, Bassel Barhoum, Katja Burian, Silvan Elias Langhorst, Janina Meißner, Barbara Streckenbach, Avinash Mohnish Suntah, Jörg Richter, Felicita Heidler, Uwe Klaus Zettl

**Affiliations:** ^1^ Neuroimmunology Section, Department of Neurology Rostock University Medical Center Rostock Germany; ^2^ Department of Neurology Ecumenic Hainich Hospital gGmbH Mühlhausen Germany; ^3^ Faculty of Health Sciences University of Hull Hull UK

**Keywords:** anxiety, comorbidities, depression, medication adherence, multiple sclerosis, personality traits, polypharmacy, temperament and character

## Abstract

**Background:**

Multiple sclerosis (MS) is an immune‐mediated disease of the central nervous system that typically requires lifelong treatment. The management of MS relies on a multifaceted approach that includes disease‐modifying therapy along with the treatment of acute relapses, MS‐related symptoms, and comorbid conditions. Medication adherence is crucial for achieving favorable treatment outcomes, but non‐adherence remains a prevalent issue. The aim of this study was to explore how individual personality traits and other factors contribute to suboptimal adherence in people with MS (pwMS).

**Methods:**

A total of 397 pwMS were included in this cross‐sectional study. Personality traits were assessed through patient questionnaires using the NEO Five‐Factor Inventory and the Temperament and Character Inventory‐Revised. Additionally, the pwMS were screened for symptoms of anxiety and depression. The patients were divided into two groups based on their self‐reported adherence to prescribed and/or non‐prescribed medication. These groups were compared regarding psychometric, sociodemographic, clinical, medication, and vaccination‐related data.

**Results:**

A subset of the pwMS (19.9%, *n* = 79) stated that they skip or miss a medication at least once a month. On average, these patients were significantly younger, had lower disability scores and a lower disease severity than the adherent pwMS. The non‐adherent patients also had significantly lower scores for self‐directedness and compassion as well as higher scores for shyness, self‐forgetfulness and anxiety. A higher proportion of non‐adherent than adherent pwMS were diagnosed with depression (24.1% vs. 14.5%). Dimethyl fumarate and interferon beta‐1b were prescribed over two times more frequently in the non‐adherent pwMS group.

**Conclusion:**

This study shows that personality traits are related to medication use in pwMS. Together with clinical‐demographic factors, they contributed to the prediction of non‐adherence in exploratory multivariable analyses. Individual patient characteristics should therefore be considered in the identification of pwMS who may have difficulties adhering to their treatment. This would facilitate targeted discussions about concerns such as side effects and the provision of support to improve medication management.

## Graphical abstract

This cross‐sectional study investigated the role of personality traits in medication non‐adherence among patients with multiple sclerosis. Non‐adherence, reported by 19.9% of the patients, was linked to younger age, lower disease severity, and distinct psychological profiles, including lower self‐directedness, higher shyness, and higher anxiety scores. These findings support integrating personality and clinical factors to identify at‐risk patients and improve individualized, patient‐centered care.

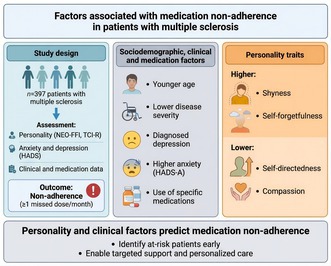

**Abbreviations**
AUC: area under the curve
C: cooperativeness
CIS: clinically isolated syndrome
DMT: disease‐modifying therapy
EDSS: Expanded Disability Status Scale
FDR: false discovery rate
HA: harm avoidance
HADS: Hospital Anxiety and Depression Scale
IQR: interquartile range
LASSO: least absolute shrinkage and selection operator
MS: multiple sclerosis
MSSS: Multiple Sclerosis Severity Score
NEO‐FFI: NEO Five‐Factor Inventory
NS: novelty seeking
OR: odds ratio
P: persistence
PPMS: primary progressive multiple sclerosis
pwMS: patients with multiple sclerosis
RD: reward dependence
RRMS: relapsing–remitting multiple sclerosis
SARS‐CoV‐2: severe acute respiratory syndrome coronavirus 2
SD: self‐directedness
SPMS: secondary progressive multiple sclerosis
ST: self‐transcendence
TCI‐R: Temperament and Character Inventory‐Revised

## 1 Introduction

Multiple sclerosis (MS) is a chronic immune‐mediated disease that affects the central nervous system, involving both inflammatory and neurodegenerative components [1]. It is the leading cause of non‐traumatic neurological disability in young adults and affects 2.9 million people worldwide [2]. Approximately 85%–90% of patients with MS present initially with the relapsing–remitting form (RRMS), featuring acute neuroinflammatory episodes, followed by either full or partial recovery [3, 4]. In contrast, progressive forms of MS are characterized by chronic low‐grade inflammation and gradual neurodegeneration [1]. Most patients with RRMS eventually convert to a progressive course over time, which is called secondary progressive MS (SPMS) [5]. Primary progressive MS (PPMS) occurs in 10%–15% of patients, with a continuous accumulation of neurological disability from disease onset, typically without relapses [5]. Symptoms experienced by people with MS (pwMS) include restricted mobility caused by paresis and spasticity, vision problems, bladder and bowel difficulties, sexual dysfunction, cognitive impairment, depression, and fatigue [6, 7].

There is still no cure for MS, but numerous disease‐modifying therapies (DMTs) have been approved that effectively reduce disease activity, prevent new lesions, decrease relapse frequency, and slow disability progression [8, 9, 10, 11]. The number of DMTs for MS continues to grow, broadening the decision‐making process regarding the optimal treatment for a patient. Beyond DMTs, high‐dose glucocorticoids are typically used to treat acute relapses, while a broad spectrum of medications is available to manage the diverse symptoms of MS [12]. As comorbid conditions are common in pwMS [13], they often necessitate medical treatment as well. Many patients also use self‐medication with over‐the‐counter drugs, vitamins, mineral supplements, and other complementary or alternative medicines [14, 15, 16]. As a result, polypharmacy is prevalent among pwMS, with studies indicating that between 14% and 76.5% of pwMS use at least five different medications concurrently [17, 18].

Adherence to treatment is a key factor in achieving optimal therapeutic outcomes and improving the overall quality of life in pwMS. The World Health Organization defines adherence as the degree to which an individual's actions, such as taking medication, following a diet, and/or making lifestyle changes, align with the recommendations provided by a healthcare professional [19]. Adherence is especially crucial for the effectiveness of DMTs in treating MS [20]. Adherence to DMTs has been linked to a lower risk of relapses and reduced disability progression [21, 22]. For instance, the risk of experiencing a severe relapse was 12.4% in adherent pwMS as compared to 19.9% in non‐adherent patients [23]. A recent study reported a high and stable self‐reported adherence to DMTs over time, regardless of the administration route, but the median time to discontinuation was lower in pwMS on injection therapies (30 months) than on oral or infusion therapies (36 months) [24]. Additionally, it has been observed that adherence is generally better in pwMS than in those with other chronic conditions, with optimal adherence being observed in 76% of MS patients compared to ~50% of patients with cardiovascular and psychiatric diseases [25]. Within the MS cohort, adherence was shown to be higher for DMTs than for chronic‐use non‐MS medications [26]. Adherence to medication corresponds with lower rates of hospital stays and absences from work as well as lower overall costs for patient care [21]. Adherence is thus important not only for DMTs but for the entirety of all medications used.

Personality traits are relatively enduring patterns of thoughts, feelings and behaviors that reflect the tendency to respond in certain ways under certain circumstances [27]. MS patients were found to be more neurotic and less extroverted compared to age and sex‐matched healthy controls [28, 29]. Moreover, higher harm avoidance and lower self‐directedness were observed in pwMS compared to controls [30, 31], with these traits correlating with increased fatigue in pwMS [32]. However, there is so far limited data on the impact of personality traits on medication adherence and whether polypharmacy predisposes to non‐adherence in pwMS. In a study comprising 56 pwMS, Bustos et al. have assessed the relationship between self‐reported medication adherence and personality styles. They showed that pleasure‐enhancing, pain‐avoiding and confident/asserting personalities were associated with an increase in adherence to drug treatment, while the presence of other‐nurturing, feeling‐guided and dutiful/conforming personalities was associated with lower adherence [33]. In an earlier study by Bruce et al., the association between personality and retrospective self‐reported adherence to glatiramer acetate or interferon beta was investigated in 55 patients with RRMS [34]. The study revealed that poor adherence correlated with lower levels of conscientiousness as well as higher levels of neuroticism, openness, and anxiety. The association between conscientiousness and treatment adherence was also demonstrated in a study with 112 pwMS [35], but not in a study with 41 RRMS cases [36]. The association with neuroticism is consistent with a study showing that pwMS with Type D personality, a combination of higher neuroticism and greater social discomfort, which was present in 16% of 230 pwMS, reported significantly worse adherence to their prescribed MS medication [37]. Higuera et al. found that pwMS with a diagnosed depression were 5.5% less likely to be adherent to their DMTs compared to the reference group [38]. A range of studies in chronic conditions other than MS has likewise investigated the impact of personality traits on medication adherence. For instance, neuroticism was associated with poor adherence in patients with type 2 diabetes mellitus [39], whereas conscientiousness was linked to adherence in individuals with cardiovascular disease [40]. Adherence may be particularly challenging for patients with polypharmacy, as a higher number of prescribed medications and greater regimen complexity were found to have a negative effect on adherence in some studies [41].

In the present study, we sought to investigate the association between personality traits and self‐reported medication use in pwMS. We further aimed to examine the impact of anxiety and depression on medication adherence. Based on the evidence from the aforementioned studies, we hypothesized that traits reflecting greater self‐regulation, goal orientation, and reliability, such as higher conscientiousness and self‐directedness, would be associated with better medication adherence. Conversely, traits related to emotional distress or avoidance, such as higher neuroticism, harm avoidance, and anxiety, were expected to be associated with poorer adherence. In addition to the patients' psychometric profiles, we explored sociodemographic variables, clinical characteristics (including comorbidities), medication data (covering both prescribed and non‐prescribed drugs), and vaccination‐related data as potential determinants of non‐adherence. Overall, this study aimed to advance our understanding of factors influencing medication adherence in pwMS to inform more personalized patient care strategies.

## 2 Materials and Methods

### 2.1 Patient Enrollment and Inclusion Criteria

**FIGURE 1:**
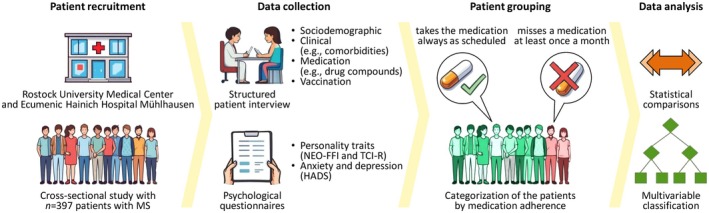
Overview of the study. In this bicentric cross‐sectional study, a total of 397 patients with MS who were taking at least one medication were included. Different types of data were collected through patient interviews, a review of medical records and clinical examinations. Established self‐report questionnaires were used to assess personality traits as well as symptoms of anxiety and depression. The patients were divided into two groups based on whether they reported not taking a medication at least once per month. Finally, the groups were compared to identify risk factors for medication non‐adherence. HADS, Hospital Anxiety and Depression Scale; MS, multiple sclerosis; *n*, number of patients; NEO‐FFI, NEO Five‐Factor Inventory; TCI‐R, Temperament and Character Inventory‐Revised.

This bi‐center, cross‐sectional and observational study was conducted between June 2019 and January 2022 [42, 43, 44]. A total of 461 pwMS from the Department of Neurology at the Rostock University Medical Center in Rostock (Germany, population size: ~210,000 residents) and the Department of Neurology at the Ecumenical Hainich Hospital in Mühlhausen (Germany, population size: ~37,000 residents) were asked to participate voluntarily. These medical centers have dedicated outpatient and inpatient units, serving MS patients from two broad regional areas in eastern Germany. The inclusion criteria consisted of patients aged 18 years or older with a confirmed diagnosis of MS or a clinically isolated syndrome (CIS) according to the revised McDonald criteria from 2017 [45]. A total of 57 patients were not included in the study due to unwillingness to participate after being informed about the study (*n* = 33), lack of time (*n* = 18), diagnosis other than CIS or MS (*n* = 3) or inability to provide informed consent or complete the questionnaires (*n* = 3). Of the remaining 404 patients, we excluded patients who were not taking any medication (*n* = 7). Thus, 397 pwMS were included for the present study (Figure 1).

### 2.2 Collection of General Patient Data

Basic patient data were obtained through anamnesis, a review of medical records, clinical examination, and a structured patient interview. By this means, sociodemographic, clinical, medication, and vaccination‐related data were collected, as detailed below.

Sociodemographic information included age, sex, partnership status (single or living in a partnership), educational background, employment status, disability retirement status, number of siblings and children, smoking status (current, former or never), and place of residence. The latter was classified into four categories: rural community (< 5000 residents), small town (5000–19,999 residents), medium‐sized town (20,000–99,999 residents), and city (> 100,000 residents).

Clinical data included MS disease course, degree of disability (assessed using Kurtzke's Expanded Disability Status Scale (EDSS) [46]), disease duration (measured in years since the initial diagnosis of MS or CIS) and comorbidities. Comorbidity was defined as any additional disease that coexists in an individual with MS, independently of the disease (i.e., not as a symptom or complication of MS), following international recommendations [13, 47]. The pwMS were stratified by the type of care they received, with inpatients being treated for acute disease activity, disease progression or adverse drug events over several days and outpatients attending regular appointments for the administration of DMTs via intravenous infusions or routine check‐ups. The global Multiple Sclerosis Severity Score (MSSS) was calculated for each patient as a measure of disease severity, adjusting disability (i.e., EDSS score) by disease duration [48].

The medication data encompassed all drugs that were taken by the patients at the time of data collection. This included both prescribed medications as well as non‐prescribed self‐medications, such as over‐the‐counter medicines, vitamins, and other dietary supplements. The total number of medications used was categorized according to the treatment objective: DMTs for MS, medications to treat MS‐related symptoms, and medications for comorbidities and other conditions. Additionally, we distinguished between medications that were taken on a long‐term basis and those used on demand. Furthermore, we recorded the number of previous DMT discontinuations. The pwMS were categorized into those with and without polypharmacy based on the number of drugs they were taking. In accordance with the most common definition, polypharmacy was defined as the concurrent use of at least five medications [49].

Regarding vaccination data, the pwMS were asked whether or not they were willing to receive all standard vaccinations, defined as those recommended by the Standing Committee on Vaccination in Germany [50]. The willingness to get vaccinated against infectious diseases was included in our study, as infections pose a significant risk for pwMS [51, 52] and the readiness for prophylactic measures is crucial for preventing adverse health outcomes [42]. The pwMS were also asked whether they get vaccinated against influenza annually. Moreover, a subset of the patients (*n* = 191) was surveyed between October 2021 and January 2022 to ask whether they had been vaccinated against the severe acute respiratory syndrome coronavirus type 2 (SARS‐CoV‐2).

### 2.3 Evaluation of Personality Traits and Psychological Symptoms

The patients were asked to complete self‐report questionnaires to assess personality traits and to measure symptoms of anxiety and depression.

The NEO Five‐Factor Inventory (NEO‐FFI) was used to assess five main personality dimensions (neuroticism, extraversion, openness, conscientiousness and agreeableness). The NEO‐FFI consists of 60 self‐descriptive statements (12 items for each dimension), each rated on a 5‐point Likert‐type scale (0 = “strongly disagree” to 4 = “strongly agree”) [53, 54, 55]. For each NEO‐FFI dimension, a cumulative score ranging from 0 to 48 was calculated. Schwartz et al. demonstrated in a large cohort of pwMS (*n* = 419) that self‐report ratings had acceptable levels of internal consistency for all NEO‐FFI domains (Cronbach's *α* ≥ 0.71) [56].

Additionally, we used the Temperament and Character Inventory‐Revised (TCI‐R), which is a commonly used personality assessment tool [57, 58]. We used the German version with 240 items and a bivariate response model [59, 60]. The TCI‐R was designed to measure four temperament dimensions (novelty seeking, harm avoidance, reward dependence, and persistence) as well as three character dimensions (self‐directedness, cooperativeness, and self‐transcendence). Whereas temperament is thought to reflect genetic influences on personality, character is regarded as shaped by environmental and cultural learning [61]. Cronbach's *α* estimates varied from 0.57 for persistence to 0.84 for harm avoidance, self‐directedness, and cooperativeness within a German normative sample [60].

Symptoms of anxiety and depression were assessed using the Hospital Anxiety and Depression Scale (HADS), a screening tool that has been developed to identify emotional disorders in non‐psychiatric patients within a hospital environment. The HADS consists of 14 items (7 items related to anxiety and 7 items related to depression), which are rated on a scale from 0 to 3 [62]. Thus, the maximum score for each subscale (anxiety and depression) is 21. For detecting major depressive disorder, a score of ≥ 8 on the depression scale gave 82% sensitivity and 74% specificity, and for detecting generalized anxiety disorder, a score of ≥ 8 on the anxiety scale gave 78% sensitivity and 74% specificity [63]. Marrie et al. reported good internal consistency (Cronbach's *α* ≥ 0.82) and good test–retest reliability (intraclass correlation coefficient: 0.83) for the two HADS subscales in pwMS [64].

### 2.4 Assessment of Medication Adherence

Non‐adherence was defined as missing one or more doses of any medication at least once per month. We assessed medication adherence based on patient self‐report. Accordingly, the pwMS were asked whether they missed taking at least one medication as scheduled once a month or once a week. Patients who reported taking all their medications regularly or missing doses less than once a month were classified as adherent. Those who reported missing at least one dose of any medication at least once a month were classified as non‐adherent. No distinction was made regarding which specific medications were missed or whether non‐adherence was intentional or unintentional. Non‐adherence could be either partial or complete. Furthermore, the pwMS were asked to specify the reasons for any missed doses. They were allowed to report multiple reasons in free‐text format. The responses were subsequently grouped into categories for the analysis.

### 2.5 Statistical Analysis and Modeling

The data were organized and prepared using Microsoft Excel 2010 and SPSS version 29.0.1.1, while the statistical analyses were conducted using SPSS and R version 4.1.2. Descriptive statistics were calculated as means, medians and interquartile ranges (IQRs, 25th and 75th percentiles) for numerical variables and as counts and percentages for categorical variables. Shapiro–Wilk tests were used to assess normality for continuous data. Comparisons of adherent and non‐adherent pwMS regarding sociodemographic, clinical, medication‐related, vaccination‐related and psychometric data were performed using Mann–Whitney *U* tests for numerical variables and chi‐squared tests or Fisher's exact tests for categorical variables. Effect sizes were calculated as absolute rank‐biserial correlations for Mann–Whitney *U* tests, Cramér's *V* for chi‐squared tests and Phi coefficients for Fisher's exact tests, with values around 0.1, 0.3 and 0.5 generally corresponding to small, medium and large effects, respectively [65]. As the number of pwMS with particular comorbidities or receiving specific drug compounds was low, one‐tailed Fisher's exact tests with mid‐p adjustment (R package exact2x2) were used. The mid‐p adjustment was chosen to reduce the conservativeness of exact testing in sparse contingency tables, and the one‐tailed approach was chosen because these exploratory analyses were specifically intended to identify comorbidities and drug compounds that were overrepresented among non‐adherent patients. All statistical analyses were based on valid data only, thus cases with missing data were omitted. The significance level was set at *α* = 0.05. Although the analyses were exploratory, we also adjusted the *p* values for multiple testing using the false discovery rate (FDR) [66]. Pie charts and bar charts were used to visualize the prevalence and reasons for medication non‐adherence.

A multivariable classification analysis was performed to identify risk factors that are predictive of medication non‐adherence in pwMS. A total of 475 variables were considered, including 32 variables related to sociodemographics, clinical status (using two dummy variables for disease course SPMS and PPMS), medication use and vaccinations, 43 variables for psychometric data, 101 variables for comorbidities and 299 variables for drug compounds used. The variables were not standardized, and ordinal variables were treated as numeric. The classification task was performed using the XGBoost algorithm (R package xgboost) [67], which works by training multiple decision trees on subsets of the data. The predictions of each tree are then combined to produce the final prediction. XGBoost is robust to collinearity due to its tree‐based structure, non‐linear decision boundaries and implicit feature selection during training. Moreover, it natively handles missing values by learning default split directions during tree construction. The data was randomly divided into an 80% training set and a 20% test set. A 5‐fold cross‐validation was first performed on the training set to determine the optimal number of boosting iterations, with early stopping triggered if the validation log loss did not improve for 10 rounds. The final XGBoost model was then trained on the entire training set using this optimal number of iterations, with a maximum tree depth of 3, a learning rate of eta = 0.1, a binary logistic objective and log loss as the evaluation metric. The learning curves for the training and validation folds were plotted to illustrate the model's learning dynamics. The precision‐recall curve (R package PRROC) and model accuracy were then computed on the independent test set. Additionally, we calculated how much each feature contributes to improving the model's predictions (i.e., gain). Finally, the first XGBoost tree was visualized as an illustrative example to provide insight into how the model makes initial decisions. This single tree was not intended to represent the full ensemble or to provide a stable clinical decision rule. The visualization was generated using the supertree package (version 0.5.5) for Python (version 3.11.5) in Jupyter Notebook (version 7.3.2).

To complement the XGBoost analysis and provide interpretable effect estimates, medication adherence was analyzed as a binary outcome using penalized multivariable regression. This analysis considered the sociodemographic, clinical, medication, vaccination‐related, and psychometric data. However, comorbidities, drug compounds, and SARS‐CoV‐2 vaccination status were omitted because the penalized regression model cannot handle missing values and because including all variables would greatly exceed the sample size, potentially compromising model stability. Variable selection was performed using least absolute shrinkage and selection operator (LASSO) regression with a logistic link function (R package lars). The optimal regularization parameter was chosen by minimizing the prediction error in 10‐fold cross‐validation. Predictors with non‐zero coefficients at the optimal regularization step were retained. To obtain classical effect estimates for exploratory purposes, a multivariable logistic regression model was subsequently fitted including only the variables selected by LASSO. The regression coefficients were then used to calculate odds ratios (ORs), which were visualized in a forest plot.

## 3 Results

### 3.1 Overview of the Patient Cohort

A total of 397 pwMS were included in this study, of whom 68.8% (*n* = 273) were women (Table 1). The median age of the patients was 49 years (IQR: 38–58) and the median duration of school attendance was 10 years (IQR: 10–12). The majority of the pwMS were skilled workers (60.2%, *n* = 239), followed by those with higher education at a technical college or university (36.2%, *n* = 144). Almost half of the patients (46.6%, *n* = 185) were part‐time or full‐time employed, while 34.8% (*n* = 138) received a disability pension. A large number of patients (39.0%, *n* = 155) lived in rural communities, followed by cities (26.7%, *n* = 106), medium‐sized towns (19.1%, *n* = 76) and provincial towns (15.1%, *n* = 60). Most of the pwMS (74.8%, *n* = 297) were living in a partnership. The mean number of children and siblings was 1.2 and 1.6, respectively. The majority of the patients were non‐smokers (69.9%, *n* = 274), of whom 31.9% (*n* = 125) were former smokers.

**TABLE 1:** Overview of the patient cohort and comparison by medication adherence.

Characteristic	Total (*n* = 397)	Adherent (*n* = 318)	Non‐adherent (*n* = 79)	Effect size	*p*
**Sociodemographic data**					
Age (years), median (IQR)	49 (38–58)	50.5 (38.3–58.8)	45 (34.0–56.5)	0.18	**0.013** ^U^
Sex, *n* (%)				0.04	0.501^Fi^
Women	273 (68.8)	216 (67.9)	57 (72.2)		
Men	124 (31.2)	102 (32.1)	22 (27.8)		
School years, median (IQR)	10 (10–12)	10 (10–12)	10 (10.0–10.5)	0.04	0.503^U^
Educational level, *n* (%)				0.08	0.516^chi^
No training	14 (3.5)	9 (2.8)	5 (6.3)		
Skilled worker	239 (60.2)	193 (60.7)	46 (58.2)		
Technical college	72 (18.1)	58 (18.2)	14 (17.7)		
University	72 (18.1)	58 (18.2)	14 (17.7)		
Employed, *n* (%)	185 (46.6)	145 (45.6)	40 (50.6)	0.04	0.451^Fi^
Disability retired, *n* (%)	138 (34.8)	110 (34.6)	28 (35.4)	0.01	0.896^Fi^
Place of residence, *n* (%)				0.21	**0.001** ^chi^ *
Rural community	155 (39.0)	127 (39.9)	28 (35.4)		
Provincial town	60 (15.1)	41 (12.9)	19 (24.1)		
Medium‐sized town	76 (19.1)	54 (17.0)	22 (27.8)		
City	106 (26.7)	96 (30.2)	10 (12.7)		
Living in a partnership, *n* (%)	297 (74.8)	239 (75.2)	58 (73.4)	0.02	0.773^Fi^
No. of children, median (IQR)	1 (0–2)	1 (0–2)	1 (0–2)	0.02	0.769^U^
No. of siblings, median (IQR)	1 (1–2)	1 (1–2)	1 (1–2)	0.10	0.148^U^
Smoking status, *n* (%)				0.03	0.829^chi^
Current smoker	118 (30.1)	92 (29.4)	26 (32.9)		
Former smoker	125 (31.9)	101 (32.3)	24 (30.4)		
Never smoker	149 (38.0)	120 (38.3)	29 (36.7)		
**Clinical data**					
Disease course, *n* (%)				0.07	0.391^chi^
CIS/RRMS	273 (68.8)	214 (67.3)	59 (74.7)		
SPMS	93 (23.4)	77 (24.2)	16 (20.3)		
PPMS	31 (7.8)	27 (8.5)	4 (5.1)		
Disease duration (years), median (IQR)	10 (5–19)	11 (5–19)	9 (5–18)	0.03	0.651^U^
EDSS score, median (IQR)	3 (2–5)	3.5 (2.0–5.5)	2 (1.3–4.3)	0.20	**0.006** ^U^
Global MSSS, median (IQR)	4.5 (2.3–6.6)	4.7 (2.7–6.8)	3.4 (1.3–6.1)	0.22	**0.003** ^U^ *
Medical center, *n* (%)				0.13	**0.012** ^Fi^
Rostock	196 (49.4)	167 (52.5)	29 (36.7)		
Mühlhausen	201 (50.6)	151 (47.5)	50 (63.3)		
Patient care, *n* (%)				0.10	0.055^Fi^
Outpatient	304 (76.6)	237 (74.5)	67 (84.8)		
Inpatient	93 (23.4)	81 (25.5)	12 (15.2)		
No. of comorbidities, median (IQR)	2 (0–3)	2 (0.3–3.0)	2 (0–3)	0.03	0.717^U^
**Medication data**					
No. of all drugs taken, median (IQR)	5 (3–7)	4 (3–7)	5 (3–7)	0.01	0.882^U^
Polypharmacy, *n* (%)	199 (50.1)	158 (49.7)	41 (51.9)	0.02	0.802^Fi^
DMT use, *n* (%)	313 (78.8)	250 (78.6)	63 (79.7)	0.01	0.879^Fi^
No. of prior DMT discontinuations, median (IQR)	1 (0–2)	1 (0–2)	1 (0–2)	0.03	0.705^U^
No. of symptomatic drugs taken, median (IQR)	1 (0–3)	1.5 (0–3)	1 (1–3)	0.01	0.893^U^
No. of comorbidity drugs taken, median (IQR)	2 (1–3)	2 (1–3)	2 (1–4)	0.00	0.957^U^
No. of prescription drugs taken, median (IQR)	3 (2–6)	3 (2–5)	4 (2–6)	0.01	0.867^U^
No. of non‐prescription products taken, median (IQR)	1 (0–2)	1 (0–2)	1 (0–1)	0.03	0.618^U^
No. of long‐term drugs taken, median (IQR)	4 (2–6)	4 (2–6)	4 (2–6)	0.02	0.742^U^
No. of on‐demand drugs taken, median (IQR)	0 (0–1)	0 (0–1)	0 (0–1)	0.08	0.197^U^
**Vaccination data**					
Willing to receive standard vaccinations, *n* (%)	292 (73.6)	230 (72.3)	62 (78.5)	0.06	0.319^Fi^
Gets vaccinated against influenza annually, *n* (%)	137 (34.5)	118 (37.1)	19 (24.1)	0.11	**0.034** ^Fi^
SARS‐CoV‐2 vaccination status, *n* (%)				0.10	0.240^Fi^
Vaccinated	162 (84.8)	125 (86.8)	37 (78.7)		
Not vaccinated	29 (15.2)	19 (13.2)	10 (21.3)		

*Note:* This table presents sociodemographic, clinical, medication and vaccination data for the total patient cohort and the two subgroups. The patients with MS were classified as non‐adherent when they stated in the questionnaire that they skip or miss a medication at least once a month. The vaccination status against SARS‐CoV‐2 was assessed in a subset of patients ~1 year after the vaccination campaign began. Cases with missing data were omitted from the statistical analyses (*n* = 5 for smoking status and *n* = 206 for SARS‐CoV‐2 vaccination status). Significant differences (*p* < 0.05) are indicated in bold. *FDR‐adjusted *p* value < 0.05, ^chi^chi‐squared test, ^Fi^Fisher's exact test, ^U^Mann–Whitney *U* test.

Abbreviations: CIS, clinically isolated syndrome; DMT, disease‐modifying therapy; EDSS, Expanded Disability Status Scale; FDR, false discovery rate; IQR, interquartile range; MS, multiple sclerosis; MSSS, Multiple Sclerosis Severity Score; *n*, number of patients; PPMS, primary progressive multiple sclerosis; RRMS, relapsing–remitting multiple sclerosis; SARS‐CoV‐2, severe acute respiratory syndrome coronavirus 2; SPMS, secondary progressive multiple sclerosis.

A total of 273 patients (68.8%) had a diagnosis of RRMS (*n* = 253) or CIS (*n* = 20). More than one fifth of the patients (23.4%, *n* = 93) had SPMS, and 31 patients (7.8%) had PPMS. The study population had a median EDSS score of 3 (IQR: 2–5) and a median disease duration of 10 years (IQR: 5–19 years). The disease severity was rated with a median global MSSS of 4.5 (IQR: 2.3–6.6). A high proportion of the pwMS were treated as outpatients (76.6%, *n* = 304). Most of the patients suffered from one (23.4%, *n* = 93) or more (51.1%, *n* = 203) comorbidities in addition to MS.

The average number of drugs taken per patient was 5.0, and 199 (50.1%) of the pwMS were affected by polypharmacy. The majority of patients (78.8%, *n* = 313) were treated with a DMT. Medication for managing MS‐related symptoms was used by 73.3% (*n* = 291) of the pwMS, while medication for other conditions was used by 78.3% (*n* = 311). The average number of prescribed and non‐prescribed products was 3.9 and 1.1, respectively.

When asked about their willingness to receive standard vaccinations, 73.6% (*n* = 292) of the patients responded positively. Over one third of the patients stated that they receive an influenza vaccination every year (34.5%, *n =* 137), while 84.8% (*n* = 162 out of 191 pwMS surveyed) had been vaccinated against SARS‐CoV‐2.

### 3.2 Association of General Patient Data With Medication Adherence

**FIGURE 2:**
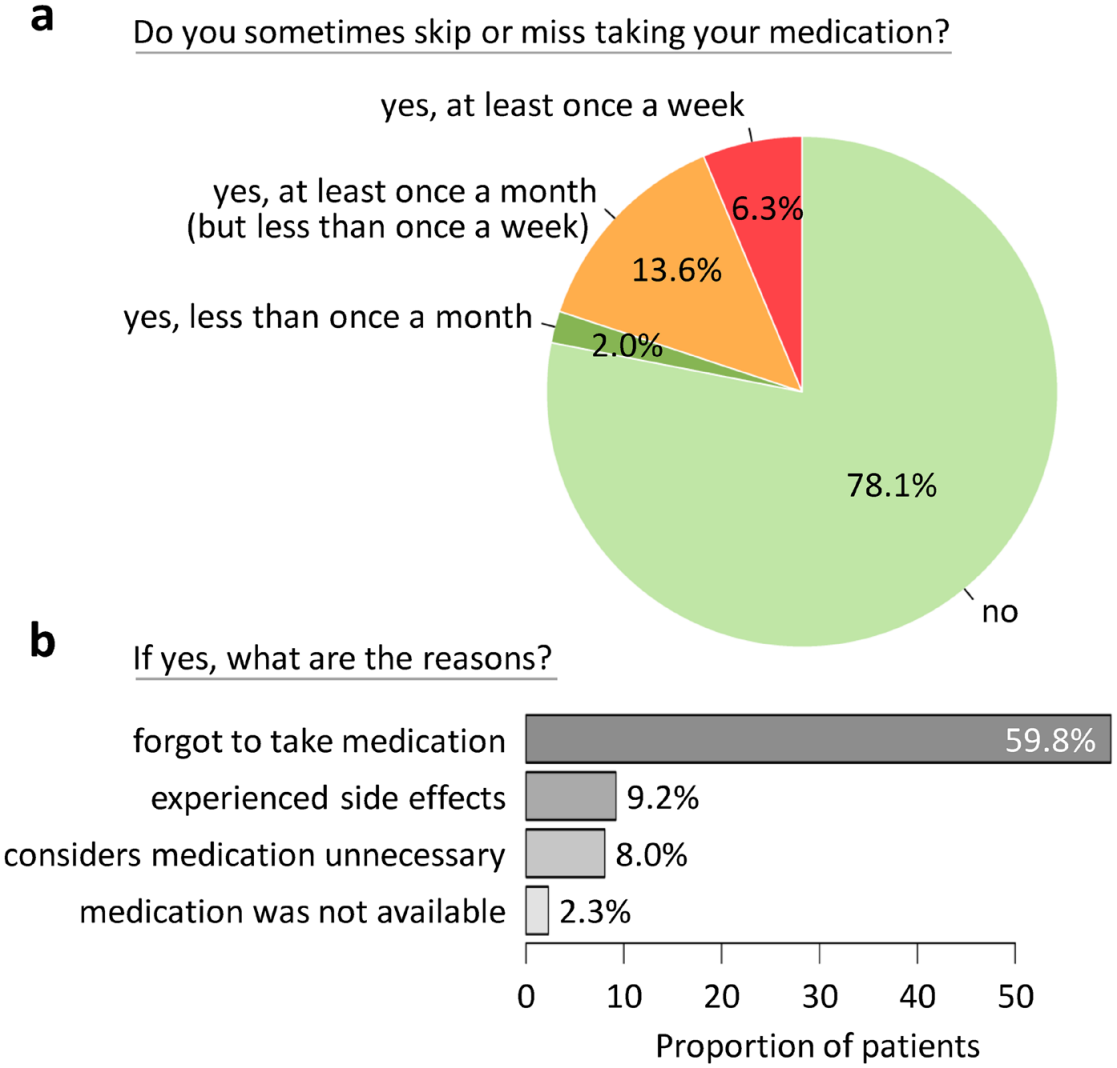
Prevalence and reasons for medication non‐adherence in patients with MS. A total of 397 MS patients who were taking at least one medication were included in this study. (a) Pie chart depicting the prevalence of medication non‐adherence, broken down by different time intervals. The majority of MS patients (78.1%, *n* = 310) reported taking their medication as scheduled. (b) Frequency of reasons for medication non‐adherence among the remaining patients (21.9%, *n* = 87). Multiple responses were possible. MS, multiple sclerosis; *n*, number of patients.

A total of 79 patients (19.9%) were classified as non‐adherent as they admitted not taking a medication at least once a month, and 318 patients (80.1%) were considered adherent. More specifically, most pwMS stated that they take their medication regularly (78.1%, *n* = 310), while the remaining patients reported missing medication less than once a month (2.0%, *n* = 8), at least once a month (13.6%, *n* = 54), or at least once a week (6.3%, *n* = 25) (Figure 2a). Among the patients reporting missed doses, forgetfulness was the most common reason for non‐adherence (59.8%, *n* = 52), followed by side effects (9.2%, *n* = 8), while 8.0% (*n* = 7) refrained from taking a medication because they considered it unnecessary (Figure 2b).

Adherent pwMS were significantly older than non‐adherent pwMS (median age: 50.5 vs. 45 years, *p* = 0.013) and more likely to live in a city (30.2% vs. 12.7%, *p* = 0.001) (Table 1). The latter was also reflected by the fact that patients from the medical center in Mühlhausen (a medium‐sized town) were significantly more likely to be non‐adherent than patients from the center in Rostock (a city). Furthermore, adherent patients had higher median EDSS scores (3.5 vs. 2, *p* = 0.006) and MSSS scores (4.7 vs. 3.4, *p* = 0.003), with the latter difference remaining significant even after adjustment for multiple testing. A significantly higher percentage of adherent pwMS self‐reported receiving an annual influenza vaccination than non‐adherent pwMS (37.1% vs. 24.1%, *p* = 0.034). Interestingly, no significant differences were found in the medication data between adherent and non‐adherent pwMS. Polypharmacy was only slightly more common among non‐adherent pwMS than among adherent pwMS (51.9% vs. 49.7%, *p* = 0.802). Even when the medications were categorized by treatment goal, prescription situation or duration of use, the number of medications taken (and the use of at least one such medication) was not significantly associated with non‐adherence.

The non‐adherent pwMS who reported forgetfulness as the reason for not taking their medication were significantly younger (median: 39 vs. 52.5 years, *U* test *p* = 0.008), more likely to be female (80.9% vs. 59.4%, Fisher's exact test *p* = 0.044), and more often current smokers (46.8% vs. 12.5%, chi‐squared test *p* = 0.006) than the other non‐adherent pwMS. Apart from that, there were no other significant differences in the sociodemographic data between these two subgroups of non‐adherent patients.

### 3.3 Personality Traits of Adherent Versus Non‐Adherent Patients With MS

Regarding the five NEO‐FFI personality dimensions, there were no significant differences between adherent and non‐adherent pwMS in the univariable analysis (Table 2). The neuroticism scores were slightly higher in non‐adherent than in adherent patients (median: 24 vs. 22), but this difference did not reach statistical significance (*p* = 0.193).

**TABLE 2:** Psychometric data in relation to medication adherence in patients with MS.

Psychometric score	Total (*n* = 397)	Adherent (*n* = 318)	Non‐adherent (*n* = 79)	Effect size	*p* ^U^
**NEO‐FFI**					
Neuroticism	22 (18–27)	22 (18.0–26.4)	24 (19.0–27.8)	0.10	0.193
Extraversion	25 (22–28)	25 (22–28)	24 (22–29)	0.05	0.505
Openness	26 (23–29)	26 (24–29)	26 (23–28)	0.09	0.219
Agreeableness	28 (24–32)	28 (24–32)	27 (24.3–31.8)	0.04	0.599
Conscientiousness	31 (28–35)	31 (28–36)	30.5 (26.4–35.0)	0.05	0.543
**TCI‐R**					
Novelty seeking	15 (12–18)	15 (12–18)	15 (12–19)	0.02	0.846
NS1: exploratory excitability	5 (4–7)	5 (4–7)	5 (4–6)	0.07	0.380
NS2: impulsiveness	3 (2–5)	4 (2–5)	3 (2–5)	0.05	0.532
NS3: extravagance	4 (3–6)	4 (3–5)	4 (3–6)	0.04	0.611
NS4: disorderliness	2 (1–3)	2 (1–3)	2 (1–3)	0.07	0.376
Harm avoidance	18 (13–23)	18 (13–23)	19 (14–24)	0.12	0.142
HA1: anticipatory worry	5 (3–7)	5 (3–7)	5 (3–7)	0.03	0.725
HA2: fear of uncertainty	5 (3–6)	5 (3–6)	5 (4–6)	0.01	0.948
HA3: shyness with strangers	3 (2–5)	3 (2–5)	4 (2–5)	0.17	**0.033**
HA4: fatigability and asthenia	6 (4–7)	5 (4–7)	6 (5–7)	0.14	0.093
Reward dependence	19 (15–22)	19 (15–22)	18 (13–22)	0.05	0.507
RD1: sentimentality	5 (4–6)	5 (4–6)	6 (3.3–7.0)	0.01	0.874
RD2: openness to warm communication	6 (4–8)	6 (4–8)	6 (4–8)	0.03	0.712
RD3: attachment	4 (2–5)	4 (2–5)	3.5 (2–5)	0.08	0.300
RD4: dependence on approval by others	4 (3–5)	4 (3–4)	3 (3–5)	0.04	0.604
Persistence	20 (15–24)	20 (15–24)	19 (14.3–23.0)	0.03	0.677
P1: eagerness of effort	6 (5–7)	6 (5–8)	6 (5–7)	0.01	0.916
P2: work hardened	5 (4–6)	5 (4–6)	5 (3.3–6.0)	0.10	0.209
P3: ambitious	4 (3–6)	4 (3–6)	4 (2–6)	0.01	0.886
P4: perfectionist	4 (3–6)	4 (3–6)	4 (3–6)	0.04	0.628
Self‐directedness	31 (25–35)	32 (26–35)	28 (24.0–31.8)	0.26	**0.002**
SD1: responsibility	6 (5–7)	6 (5–7)	5 (4–7)	0.19	**0.019**
SD2: purposefulness	5 (3.5–5.0)	5 (4–5)	4 (3–5)	0.10	0.222
SD3: resourcefulness	4 (3–5)	4 (3–5)	4 (3–4)	0.15	0.056
SD4: self‐acceptance	8 (6–9)	8 (6–9)	7.5 (5–9)	0.19	**0.022**
SD5: enlightened second nature	9 (7–10)	9 (8–10)	8 (6.3–10.0)	0.18	**0.030**
Cooperativeness	28 (25–30)	28 (25–30)	27 (24.3–30.0)	0.14	0.086
C1: social acceptance	7 (6–8)	7 (6–8)	7 (6–7)	0.15	0.059
C2: empathy	4 (3–5)	4 (3–5)	4 (3.3–5.0)	0.04	0.639
C3: helpfulness	6 (5–7)	6 (5–7)	6 (5–7)	0.03	0.718
C4: compassion	6 (5–7)	6 (6–7)	6 (4–7)	0.15	**0.046**
C5: pure hearted conscience	5 (4–6)	5 (4–6)	5 (4.0–5.8)	0.08	0.305
Self‐transcendence	6 (4–9)	6 (4–9)	7 (5–10)	0.15	0.068
ST1: self‐forgetful	2 (1–4)	2 (1–4)	3 (2–4)	0.17	**0.035**
ST2: transpersonal identification	2 (1–3)	2 (1–3)	2 (1–3)	0.02	0.832
ST3: spiritual acceptance	2 (1–3)	2 (1–3)	2.5 (1–4)	0.14	0.072
**HADS**					
Anxiety	7 (4–9)	7 (4–9)	8 (5.0–10.3)	0.16	**0.040**
Depression	5 (3–8)	5 (2–8)	5.5 (3–8)	0.05	0.484

*Note:* Personality traits and levels of anxiety and depression were compared between adherent and non‐adherent patients. Non‐adherence was defined as missing a medication at least once a month, as assessed through a self‐report questionnaire. Cases with missing data were omitted from the statistical analyses (up to *n* = 85 for TCI‐R subdimension scores). Values are given as median (interquartile range). Significant differences (*p* < 0.05) are indicated in bold. ^U^Mann–Whitney *U* test.

Abbreviations: HADS, Hospital Anxiety and Depression Scale; MS, multiple sclerosis; *n*, number of patients; NEO‐FFI, NEO Five‐Factor Inventory; TCI‐R, Temperament and Character Inventory‐Revised.

In the TCI‐R personality assessment, non‐adherent patients had higher scores in shyness with strangers, a subscale of the temperament dimension harm avoidance (median: 4 vs. 3, *p* = 0.033), and higher scores in self‐forgetfulness, a subscale of the character dimension self‐transcendence (median: 3 vs. 2, *p* = 0.035). Conversely, several TCI‐R personality traits were found to be more pronounced in adherent pwMS. More specifically, adherent patients had significantly higher scores than non‐adherent patients in the character dimension self‐directedness (median: 32 vs. 28, *p* = 0.002) and three of its subscales: responsibility (median: 6 vs. 5, *p* = 0.019), self‐acceptance (median: 8 vs. 7.5, *p* = 0.022), and enlightened second nature (median: 9 vs. 8, *p* = 0.030). Scores for compassion, a subscale of cooperativeness, were also significantly higher in adherent patients compared to non‐adherent patients (mean: 5.8 vs. 5.3). However, the smallest *p* value was not below the threshold of 0.0012, which would have been required to remain significant after FDR adjustment, given the number of comparisons made.

The analysis of the HADS data revealed higher scores on the anxiety subscale for non‐adherent pwMS compared to adherent pwMS (median: 8 vs. 7, *p* = 0.040). However, the screening for symptoms of depression using the depression scale showed no significant difference between the two groups (Table 2).

### 3.4 Comorbidities Overrepresented in Non‐Adherent MS Patients

A total of 101 different comorbidities were recorded for the study population (Table S1). Seven of these comorbidities were more prevalent in the group of non‐adherent pwMS compared to the group of adherent pwMS, with significant raw *p* values (Table 3). Regarding neurological disorders, non‐adherent pwMS were more frequently affected than adherent pwMS by epilepsy (5.1% vs. 0.9%, *p* = 0.018) and migraine (11.4% vs. 2.8%, *p* = 0.002). Gastrointestinal disorders were also significantly more common in non‐adherent vs. adherent pwMS (Crohn's disease: 3.8% vs. 0.3%, *p* = 0.014; gastrointestinal discomfort: 16.5% vs. 8.5%, *p =* 0.024). Moreover, a higher proportion of non‐adherent pwMS were diagnosed with depression (24.1% vs. 14.5%, *p* = 0.024), which was the second most common comorbidity after hypertension (25.4%, *n* = 101 pwMS). Of the 65 pwMS who were diagnosed with depression, 58 patients (89.2%) were treated with medication for this condition. The non‐adherent patients were also more frequently affected by cardiac arrhythmia (7.6% vs. 1.3%, *p* = 0.003) and allergy (8.9% vs. 2.8%, *p* = 0.015) compared to the adherent patients, whereas there was no significant difference with regard to anxiety disorder (3.8% vs. 1.6%, *p* = 0.126).

**TABLE 3:** Comorbidities that were more common among non‐adherent patients with MS.

Comorbidity	Total (*n* = 397)	Adherent (*n* = 318)	Non‐adherent (*n* = 79)	Effect size	*p* ^Fi^
Allergy	16 (4.0)	9 (2.8)	7 (8.9)	0.12	**0.015**
Cardiac arrhythmia	10 (2.5)	4 (1.3)	6 (7.6)	0.16	**0.003**
Crohn's disease	4 (1.0)	1 (0.3)	3 (3.8)	0.14	**0.014**
Depression	65 (16.4)	46 (14.5)	19 (24.1)	0.10	**0.024**
Epilepsy	7 (1.8)	3 (0.9)	4 (5.1)	0.12	**0.018**
Gastrointestinal discomfort	40 (10.1)	27 (8.5)	13 (16.5)	0.11	**0.024**
Migraine	18 (4.5)	9 (2.8)	9 (11.4)	0.16	**0.002**

*Note:* The patients with MS were considered non‐adherent when they did not take a medication at least once a month. Listed are secondary diseases that were diagnosed significantly more frequently in non‐adherent patients. The comorbidities are presented in alphabetical order. Values are given as *n* (%). The full list of comorbidities is provided in Table S1. Bold type indicates *p* values below 0.05. ^Fi^one‐tailed Fisher's exact test with mid‐p adjustment.

Abbreviations: MS, multiple sclerosis; *n*, number of patients.

### 3.5 Drug Compounds More Frequently Used by Non‐Adherent MS Patients

The pwMS used a total of 299 different drug compounds, including those found in combination medications, over‐the‐counter drugs and dietary supplements (Table S2). A large proportion of these compounds (44.8%, *n* = 134) were each taken by only one patient. The most commonly used compound was cholecalciferol, which was taken by 206 pwMS (51.9%). Without adjusting for multiple testing, 12 drug compounds were taken significantly more often by non‐adherent than by adherent pwMS (Table 4). This list includes DMTs for MS, namely dimethyl fumarate (10.1% vs. 4.7%) and interferon beta‐1b (6.3% vs. 2.2%), but not interferon beta‐1a (11.4% vs. 11.3%). In addition, the non‐adherent pwMS more frequently used cannabis‐based products to treat symptoms of MS (cannabidiol: 15.2% vs. 8.5%; dronabinol: 16.5% vs. 8.5%) and a medication for restless legs syndrome containing a combination of levodopa and benserazide (3.8% vs. 0.6%). Selective serotonin reuptake inhibitors were also more commonly taken by non‐adherent pwMS than by adherent pwMS (paroxetine: 2.5% vs. 0.0%; sertraline: 3.8% vs. 0.6%).

**TABLE 4:** Drugs that were more commonly used by non‐adherent patients with MS.

Drug compound	Total (*n* = 397)	Adherent (*n* = 318)	Non‐adherent (*n* = 79)	Effect size	*p* ^Fi^
Benserazide	5 (1.3)	2 (0.6)	3 (3.8)	0.11	**0.031**
Cannabidiol	39 (9.8)	27 (8.5)	12 (15.2)	0.09	**0.044**
Desloratadine	2 (0.5)	0 (0.0)	2 (2.5)	0.14	**0.020**
Dimethyl fumarate	23 (5.8)	15 (4.7)	8 (10.1)	0.09	**0.043**
Dronabinol	40 (10.1)	27 (8.5)	13 (16.5)	0.11	**0.024**
Interferon beta‐1b	12 (3.0)	7 (2.2)	5 (6.3)	0.10	**0.043**
Levodopa	5 (1.3)	2 (0.6)	3 (3.8)	0.11	**0.031**
Lisinopril	2 (0.5)	0 (0.0)	2 (2.5)	0.14	**0.020**
Memantine	2 (0.5)	0 (0.0)	2 (2.5)	0.14	**0.020**
Paroxetine	2 (0.5)	0 (0.0)	2 (2.5)	0.14	**0.020**
Salbutamol	2 (0.5)	0 (0.0)	2 (2.5)	0.14	**0.020**
Sertraline	5 (1.3)	2 (0.6)	3 (3.8)	0.11	**0.031**

*Note:* The patients with MS were considered non‐adherent when they did not take a medication at least once a month. Drug compounds that occurred significantly more frequently in the medication plans of non‐adherent patients are presented in alphabetical order. For combination medications, the individual ingredients were counted. Values are given as *n* (%). The full list of drug compounds is provided in Table S2. Bold type indicates *p* values below 0.05. ^Fi^one‐tailed Fisher's exact test with mid‐p adjustment.

Abbreviations: MS, multiple sclerosis; *n*, number of patients.

### 3.6 Multivariable Models for Medication Non‐Adherence

**FIGURE 3:**
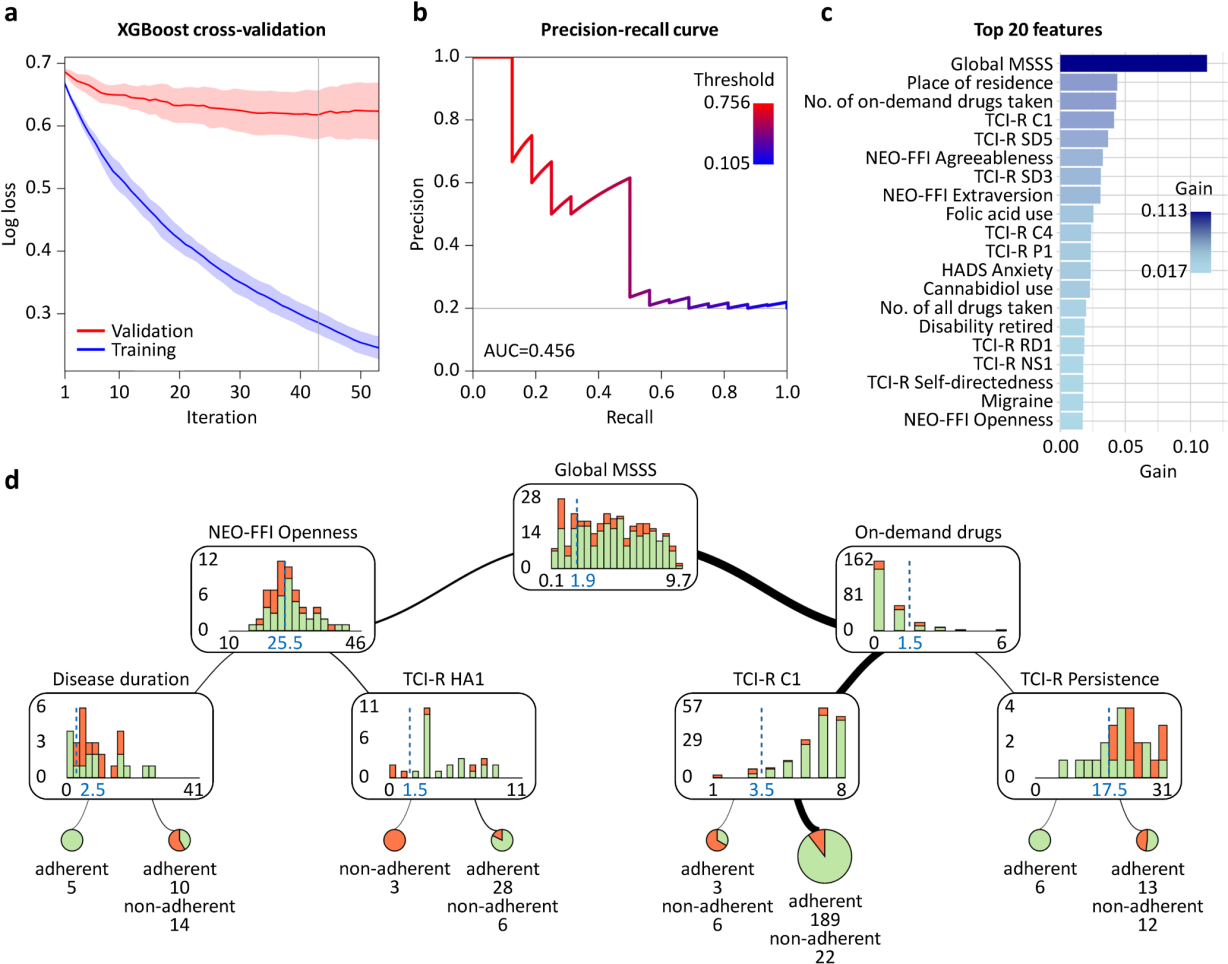
Multivariable classification of MS patients by medication adherence using XGBoost. Out of 397 patients with MS, 79 (19.9%) were considered non‐adherent as they reported missing or skipping a medication at least once a month. Sociodemographic, clinical, medication and vaccination data were collected, along with personality traits and the degree of symptoms of anxiety and depression. The data, comprising 397 patients and 475 variables, were split into an 80% training set and a 20% test set at the patient level. XGBoost was then applied for predictive modeling, with the objective parameter set to binary logistic. (a) Mean log loss across iterations from fivefold cross‐validation on the training set. Blue and red curves represent the average log loss on the training folds and the held‐out validation folds, respectively, with shaded areas indicating standard deviations. The optimal number of boosting iterations (43) used to fit the final model is shown by a gray vertical line. (b) Precision‐recall curve for the independent test set (*n* = 80; 16 non‐adherent and 64 adherent patients). The color gradient from blue to red signifies different classification thresholds. The horizontal line represents the expected precision of a random classifier, corresponding to the prevalence of the positive class (16/80 = 0.2). (c) The top 20 most important features ranked by gain, a measure of their contribution to model accuracy. The features are sorted in descending order, with bars in darker blue indicating higher relative importance. (d) The first decision tree from the trained XGBoost model, visualized using supertree. This tree is shown for illustrative purposes, as a single tree within a boosted ensemble should not be interpreted as representative of the full ensemble or as a stable stand‐alone clinical decision model, particularly in this high‐dimensional setting. Histograms show the data distribution in each node. Cut‐off values are written in blue. Pie charts beneath the leafs display the proportion of adherent and non‐adherent MS patients in the training set. The tree structure reveals that disease severity (i.e., global MSSS) served as the primary splitting criterion. As an example, all 3 patients with low MSSS, high NEO‐FFI openness and low TCI‐R HA1 scores were classified as non‐adherent. The final model achieved an accuracy of 95.6% on the training data and 82.5% on the test data using a probability threshold of 0.5. AUC, area under the curve; C, cooperativeness; C1, cooperativeness subscale “social acceptance”; HA1, harm avoidance subscale “anticipatory worry”; HADS, Hospital Anxiety and Depression Scale; MS, multiple sclerosis; MSSS, Multiple Sclerosis Severity Score; NEO‐FFI, NEO Five‐Factor Inventory; NS, novelty seeking; P, persistence; RD, reward dependence; SD, self‐directedness; TCI‐R, Temperament and Character Inventory‐Revised.

To identify possible risk factors for non‐adherence in pwMS, a multivariable classification analysis using XGBoost was first conducted on the basis of all variables (*n* = 475). The cross‐validation indicated that 43 boosting iterations was the optimal number, as the performance did not improve for 10 rounds afterwards (Figure 3a). The mean log loss decreased from 0.668 to 0.286 on the training folds and from 0.686 to 0.618 in the held‐out validation folds. The final model achieved an accuracy of 95.6% on the training data (*n* = 317) and 82.5% on the independent test data (*n* = 80). At model output values > 0.5, 50% of the non‐adherent pwMS in the test set (8/16) were correctly identified. The area under the precision‐recall curve on the test set was 0.456 (Figure 3b), indicating moderate discrimination. Among the top 20 variables with highest importance (ranked by gain) were eight TCI‐R (sub)scales (e.g., self‐directedness), three NEO‐FFI dimensions (e.g., agreeableness), the HADS anxiety scale and eight other variables (e.g., global MSSS) (Figure 3c). These variables were the most influential in the model's decision trees. In the first decision tree of the XGBoost model, the patients were initially divided into two groups based on the MSSS score and a threshold of 1.9 (Figure 3d). At deeper levels of the tree, the cases were further stratified according to scores in NEO‐FFI openness and TCI‐R (sub)scales, the number of on‐demand drugs used as well as disease duration in years. Please note that the first decision tree, as a single tree within a boosted ensemble, does not represent the full decision‐making process for classifying adherent and non‐adherent patients and should not be interpreted as a stable stand‐alone clinical decision rule. The tree is therefore shown for illustrative purposes to visualize early variable splits within the XGBoost ensemble and is not intended as a clinical decision algorithm.

**FIGURE 4:**
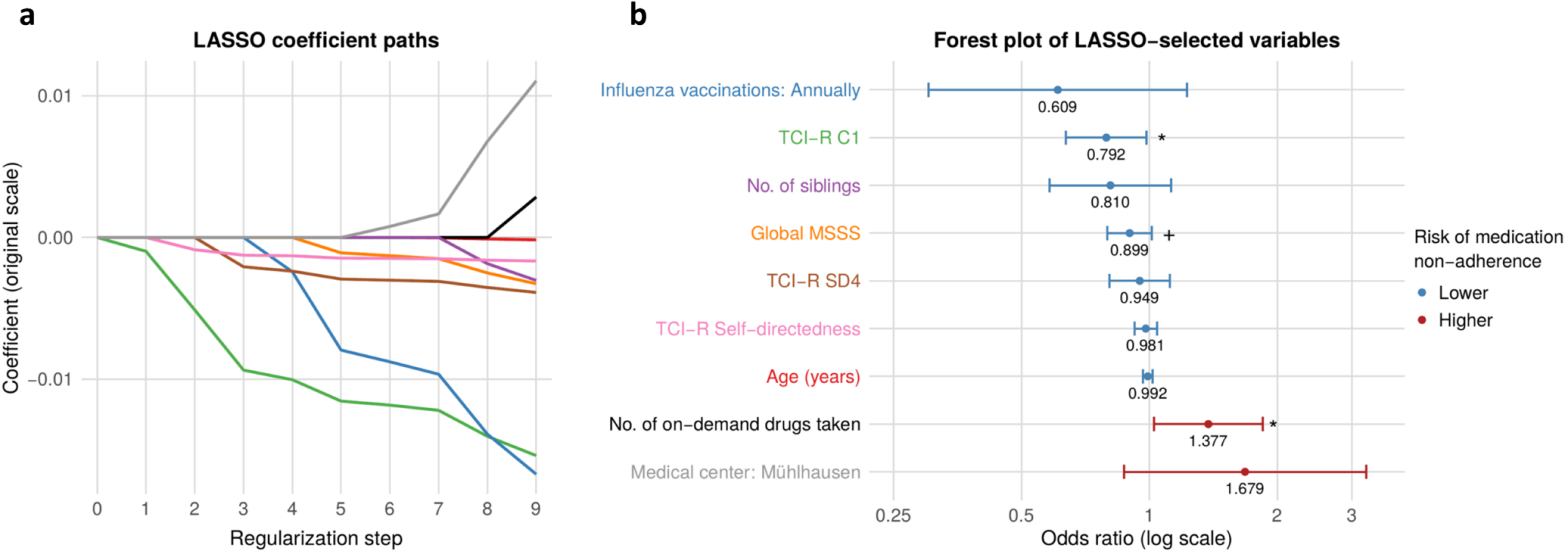
Predictors of medication non‐adherence in patients with MS identified by penalized multivariable regression. The analysis included 74 sociodemographic, clinical, medication, vaccination‐related, and psychometric variables. Comorbidities and drug compounds were not considered to reduce the dimensionality of the predictor space, and SARS‐CoV‐2 vaccination status was excluded because it was assessed only in a small subset of the patients. Cases with missing values had to be excluded, restricting the analysis to 304 MS patients with complete data. (a) LASSO coefficient paths for the nine predictors entering the model up to the optimal regularization step determined by cross‐validation. Each line represents the coefficient trajectory of one selected variable across successive regularization steps. The coefficient paths are displayed with colors that correspond to the variable labels given on the right. (b) Forest plot showing ORs and 95% confidence intervals derived from the final multivariable logistic regression model fitted using the LASSO‐selected variables. The color of the points and lines indicates the risk of medication non‐adherence, with blue representing decreased risk (OR < 1) and red representing increased risk (OR > 1). *p < 0.05, +*p* < 0.10 (*p* values are reported for descriptive purposes only, as variable selection was performed on the same dataset). C1, cooperativeness subscale “social acceptance”; LASSO, least absolute shrinkage and selection operator; MS, multiple sclerosis; MSSS, Multiple Sclerosis Severity Score; SARS‐CoV‐2, severe acute respiratory syndrome coronavirus 2; SD4, self‐directedness subscale “self‐acceptance”; TCI‐R, Temperament and Character Inventory‐Revised.

Nine predictors of medication adherence in pwMS were identified using penalized regression for variable selection followed by multivariable logistic regression (Figure 4a). Among these, TCI‐R C1 (social acceptance) and TCI‐R self‐directedness were the first two variables entering the LASSO path, underscoring the potential relevance of personality‐related factors. A higher TCI‐R C1 score was associated with a significantly reduced risk of medication non‐adherence (OR = 0.792), whereas taking a higher number of on‐demand drugs was associated with an increased risk (OR = 1.377) (Figure 4b). In addition, a higher global MSSS was associated with a lower likelihood of non‐adherence (OR = 0.899), indicating that patients with more severe disease were more likely to adhere to their medication regimen. All three of these variables also appeared in the first decision tree of the XGBoost ensemble (Figure 3d), supporting the robustness of these factors across different modeling approaches. Furthermore, MS patients who received influenza vaccinations annually had a lower risk of being non‐adherent (OR = 0.609), and patients from the Mühlhausen center were more often non‐adherent than those from Rostock (OR = 1.679). The remaining LASSO‐selected variables (age, number of siblings, the overall self‐directedness score, and the SD4 subscale score of self‐acceptance) showed more modest associations in the logistic regression model.

## 4 Discussion

Medication adherence is crucial for patient outcomes in chronic conditions like MS, where long‐term treatment is essential to reduce the risk of exacerbations, slow disease progression, and alleviate symptoms. Previous studies have shown that adherence to DMTs is associated with lower rates of MS‐related hospitalizations and relapses, resulting in reduced healthcare costs [23, 68]. However, skipping DMTs and other medication is not uncommon in pwMS and may be attributed to complex treatment regimens, polypharmacy, and patient‐specific factors. Here, we used the NEO‐FFI, TCI‐R, and HADS questionnaires to identify personality characteristics as well as psychopathological disturbances that may influence medication use in pwMS. A strength of our study is the investigation of medication adherence in a relatively large cohort of pwMS, considering the patients' entire medication and not just selected drugs. Our study provides a detailed analysis of the relationships between non‐adherence and personality traits, anxiety/depression, comorbidities, and other factors.

In our study, 80.1% of the pwMS reported that they fully adhere to their medication regimen or that they rarely miss a medication. The vast majority of the literature addresses exclusively the adherence to DMTs for MS. A systematic review from 2022, which included 24 studies, reported overall adherence rates to DMTs between 52% and 92.8% [69]. More recent studies also fall within this range [22, 26, 70], with lower adherence rates for self‐injectable and oral DMTs compared to infusion‐based DMTs [71, 72]. The variability in (non‐)adherence rates across studies can be attributed to differences in definitions and measures of adherence (e.g., via prescription claims, medication dispensations, patient diaries or questionnaires [73, 74]). In our study, adherence was assessed broadly, encompassing all medications, as we focused on the issue of polypharmacy in pwMS [43, 44]. This approach allowed us to examine general medication‐taking behavior in the context of complex medication regimens but does not allow conclusions about adherence to DMTs specifically. Furthermore, given the retrospective self‐reporting of the patients, an overestimation of medication use is likely [75]. As there is no gold standard for measuring adherence, a combination of adherence measures may be considered in future research to achieve a more accurate assessment [75, 76].

Our findings demonstrate that some TCI‐R personality traits contribute to adherence, while others are linked to suboptimal adherence in pwMS, suggesting that screening for these traits may aid in identifying non‐adherent patients. The most pronounced difference was observed in the character dimension self‐directedness, where adherent pwMS scored significantly higher than non‐adherent pwMS, both overall (median: 32 vs. 28) as well as on three of its subscales (responsibility, self‐acceptance, and enlightened second nature). Patients with high scores in these subscales tend to be responsible and feel free to choose what they want to do (responsibility), have self‐confidence and a good sense of both their strengths and limitations (self‐acceptance) and have developed a set of goal‐congruent positive habits (enlightened second nature) [77]. We are not aware of a previous study that has explored the relationship between TCI‐R personality dimensions and medication adherence in pwMS. However, similar to our study, higher self‐directedness scores were related to better adherence in patients with type 2 diabetes [78]. A study on cancer patients receiving oral chemotherapy suggested that patients with a low level of the reward dependence trait are less likely to adhere to drug therapy [79]. We could not confirm the latter association in pwMS, but the non‐adherent pwMS scored significantly higher in the TCI‐R subscales shyness with strangers and self‐forgetfulness. These patients may thus avoid social interactions [77], which could affect their treatment engagement, such as attending appointments and discussing medication, and they may be less diligent in following treatment routines. The frequent reporting of forgetfulness as a reason for missed doses may provide a descriptive link to the finding that the non‐adherent patients scored lower in self‐directedness and higher in self‐forgetfulness, traits related to self‐regulation and routine formation. When accounting for the joint effects of multiple variables, XGBoost and penalized regression both identified TCI‐R self‐directedness and social acceptance as top predictors. This convergence between the two analytical approaches reinforces the relevance of these personality factors in predicting medication adherence in our cohort. A patient's level of social acceptance might be linked to openness to guidance and trust in healthcare providers, potentially mitigating psychological reactance and thereby facilitating adherence to prescribed medication [80]. However, since we did not measure patient trust, this link remains speculative. The XGBoost model further revealed additional TCI‐R (sub)scales as predictive of medication adherence. In particular, the first decision tree indicated that persistence may contribute to non‐adherence in interaction with high disease severity and the use of multiple on‐demand drugs. While comparatively low reliability has been reported for the persistence dimension [60] and the underlying psychological mechanisms remain unclear, it is conceivable that high persistence in this context may reflect stubbornness, where patients override medical advice, or perfectionism, where patients skip doses if timing is imperfect or conflicts with other routines. Such patients may benefit from tailored counseling (e.g., encouraging timely medication catch‐up if a dose is missed) and/or a simplified regimen (e.g., consolidating on‐demand drugs). Therefore, in pwMS with pronounced personality characteristics, disease management should be closely monitored and adherence barriers addressed early.

With regard to the five NEO‐FFI personality traits, we found no significant differences between adherent and non‐adherent pwMS in the univariable analysis. However, in the multivariable analysis, NEO‐FFI openness, agreeableness, and extraversion were among the top‐ranked features by XGBoost gain. Previous research reported that high neuroticism levels and low conscientiousness levels predispose to poor treatment adherence in pwMS [34, 35, 37, 81]. Our data showed a similar but weak and non‐significant trend. A German study with 682 pwMS identified neuroticism as the strongest predictor of poor self‐management, whereas higher levels of conscientiousness positively impacted self‐management [82]. More neuroticism and less conscientiousness were found to characterize depressed/anxious pwMS compared to mentally healthy pwMS and normal controls [83]. A higher score in the neuroticism dimension has also been associated with a higher degree of disability [36]. The evidence across other diseases is inconsistent. While a study on cancer patients found that conscientiousness correlated positively and neuroticism negatively with treatment adherence [84], some studies only identified the association for conscientiousness [40, 85] and other studies found no relationship between these traits and adherence to treatment recommendations [86, 87]. Our classification analysis suggests that certain personality traits may influence adherence in subgroups of pwMS. A deeper understanding of their complex interplay with other factors may thus help develop targeted interventions to modify health‐related behaviors and improve treatment adherence in pwMS.

In our analysis, non‐adherent pwMS had significantly higher HADS anxiety scores compared to adherent pwMS (median: 8 vs. 7). The difference in HADS depression scores was not significant (median: 5.5 vs. 5), but a diagnosed depression was significantly more frequent in non‐adherent vs. adherent pwMS (24.1% vs. 14.5%). Depression and anxiety are among the most prevalent comorbid conditions among pwMS [13] and are known to influence personality traits [83, 88]. Several studies have reported higher rates of diagnosed depression [38, 69, 89] and anxiety disorders [23, 35] in pwMS who are non‐adherent to DMTs. However, this is not always reflected in studies relying on patient questionnaires to evaluate anxiety/depression. In the study by Kołtuniuk et al., no significant differences in HADS scores were observed between DMT‐adherent (*n* = 173) and non‐adherent pwMS (*n* = 53). Nonetheless, significant correlations were found between the anxiety/depression subscale scores and specific domains of treatment adherence (e.g., barriers in taking the drug regularly and side effects during treatment) [90]. In the study by Bruce et al., worse adherence to DMTs, as reported retrospectively by the pwMS, was significantly associated with increased anxiety, but not with the screening measure of depression [34], in line with our findings. The existing studies have primarily focused on DMT use. Therefore, the use of psychological symptom screening in identifying pwMS who have difficulty adhering to other medications remains largely unclear. Overall medication adherence may be improved by treating mood/anxiety disorders, providing guidance on establishing medication routines, and offering structured patient education to develop self‐management competencies [34, 82]. Mental health support may further help patients maintain consistent medication use by addressing emotional and behavioral difficulties.

We found that adherent pwMS were significantly older than non‐adherent pwMS, more likely to live in cities, had higher disability (EDSS) and disease severity (MSSS) scores, and more frequently reported receiving annual influenza vaccinations. The proportion of women was slightly, but not significantly, higher among non‐adherent pwMS. Older age and male sex are the most frequently reported factors associated with DMT adherence in pwMS [69, 89]. For example, Munsell et al. have found that being male (odds ratio: 1.20) and being older than 18–34 years (odds ratios ≥ 1.22) were significantly associated with a higher adherence to self‐injectable and oral DMTs in pwMS [91], in line with other studies [23, 38, 92]. Although not directly assessed in our study, women with MS may face greater adherence challenges due to heightened sensitivity to drug side effects, a greater propensity for cognitive fatigue [93], and additional psychosocial stressors such as caregiving responsibilities, which could affect their ability to prioritize self‐care. Possible explanations for non‐adherence in younger pwMS might be that they underestimate the importance of long‐term treatments as their symptoms are still mild or intermittent and that medication intake interferes with their everyday activities. For those patients, personalized strategies should be adopted to better integrate medication intake into daily life without disrupting work or social activities. Our finding that adherent pwMS were more likely to live in a city aligns with the study by Kołtuniuk et al., who found that a higher proportion of DMT‐adherent pwMS resided in cities (≥ 100,000 residents) compared to non‐adherent pwMS (53.5% vs. 43.4%) [94]. Higher adherence in patients living in urban vs. rural areas has also been demonstrated for other chronic diseases and linked to better access to healthcare [26, 95]. Mixed findings have been reported regarding the association between disability and non‐adherence [69, 96]. In the study by McKay et al., longer disease duration and mild disability status (EDSS < 3) were associated with non‐adherence to DMTs [97], consistent with our study. Our finding regarding influenza vaccinations reaffirms the well‐known relationship between adherence to chronic medications and adherence to preventive medicine recommendations [98]. However, in addition to patient‐related factors, treatment‐specific factors (e.g., tolerability, convenience and satisfaction) also influence medication adherence, but these were beyond the scope of the present study.

We observed similar rates of polypharmacy in both adherent and non‐adherent pwMS. The use of multiple medications may be expected to reduce adherence due to the increased complexity of the treatment regimen, making it harder for patients to manage their doses. However, Seferoğlu et al. found that patients with additional medications had better adherence to oral DMTs compared to those without [71]. They concluded that polypharmacy can influence adherence in both directions, suggesting that the use of additional medications in pwMS may enhance adherence by increasing patient awareness and routine. In our patient cohort, significantly more non‐adherent than adherent pwMS were prescribed dimethyl fumarate (taken orally twice a day) or interferon beta‐1b (administered subcutaneously every other day). Other DMTs are administered less frequently than the fumarates [8], and other interferon beta preparations can be scheduled more consistently throughout the week, facilitating adherence [21, 89, 97, 99]. The two DMTs are also less well tolerated than other DMTs [100]. However, side effects were relatively rarely mentioned by the pwMS as a reason for missing medication (9.2%), while the most common reason was simply forgetting to take it (59.8%), similar to what has been reported by others [101]. The more frequent use of certain non‐DMTs among non‐adherent pwMS corresponded with a higher prevalence of certain comorbidities in these patients. More specifically, desloratadine, which is used to treat seasonal allergies, as well as paroxetine and sertraline, which are used to treat depression, were used by a significantly higher proportion of non‐adherent than adherent pwMS, even though the number of cases was small. However, the more frequent occurrence of individual drugs in the medication plans of non‐adherent patients should not be interpreted as evidence of drug‐specific non‐adherence, as medication adherence was assessed across all medications used by the patients and therefore relates to overall medication‐taking behavior. Moreover, dosing frequency and regimen complexity were not analyzed as separate covariates in our study. Therefore, the observed differences in medication use cannot be disentangled from drug‐related factors such as dosing schedule, administration route or tolerability. Various patient‐centered solutions exist to minimize unintentional medication non‐adherence, such as using digital medication reminders (e.g., daily text‐message systems) [102, 103], streamlining medication schedules, selecting drugs with preferred routes of administration, adopting long‐acting drug delivery systems [104], and providing counseling to improve health literacy. The best approach may combine multiple strategies that are tailored to the patient's individual lifestyle, cognitive ability, and personality.

This study has several limitations that should be acknowledged. First, due to the cross‐sectional design of the study, no insight can be given into medication use patterns over time. A longitudinal analysis would therefore be beneficial in future research to better understand the drivers of non‐adherence while accounting for temporal variability. Our study was conducted in a representative patient sample that closely resembles the characteristics of pwMS in the German MS Registry [105]. On the other hand, the pwMS were recruited at specialized MS centers. Our results may thus not generalize to pwMS who are treated at outpatient neurological practices or in other countries. While the overall study population was relatively large, the number of cases in subgroups (such as those using certain drugs) was sometimes very small. The subgroup analyses of comorbidities and drug compounds were therefore conducted as directional, hypothesis‐generating screening analyses and do not allow firm conclusions. Additionally, we assessed medication adherence through retrospective patient self‐reports. Non‐adherence was defined as missing at least one dose of any medication, using an arbitrarily set threshold of once per month. The pwMS were also asked to provide reasons for missed doses, allowing us to capture perceived barriers such as considering medication unnecessary or skipping doses due to side effects. However, the use of non‐validated adherence questions is prone to biases, including recall bias and social desirability bias [76]. Moreover, the subsequent categorization into a binary outcome (adherent vs. non‐adherent) limits the robustness of the adherence measure and should be considered when interpreting the findings. Future studies should therefore employ validated and/or objective methods for measuring medication adherence [76] to verify the role of personality traits as determinants of medication (non‐)adherence in pwMS. Furthermore, we recorded all medications used by the pwMS, including symptomatic treatments and drugs prescribed for comorbidities, whereas prior research has primarily focused on adherence to DMTs [69, 89]. As we did not specifically ask the non‐adherent pwMS which medication(s) they had missed, adherence patterns for individual drug treatments could not be assessed. This means that we were unable to distinguish non‐adherence to DMTs from non‐adherence to other medications. Lastly, while we utilized different measures of personality and gathered information on education and employment, we did not adequately capture the patients' socioeconomic status (e.g., income and living conditions) and cultural background, which are known to influence psychological traits and treatment adherence [106, 107]. Further studies are also needed to confirm the use of personality assessments in combination with other patient‐related factors in predicting medication adherence in pwMS. A better understanding of the links between personality, cognition and behavior will be useful in optimizing medication adherence in pwMS, ensuring better long‐term disease control, especially for those with low disease severity who tend to be less attentive in taking their medication.

## 5 Conclusion

The treatment regimen of pwMS is often complex, which is reflected in a relatively high rate of polypharmacy. Our exploratory study provides new insights into how personality traits are associated with intentional or unintentional medication non‐adherence in pwMS, although these findings should be interpreted as hypothesis‐generating because adherence was assessed by self‐report and across all medications rather than for specific drug treatments. Our data indicate that patients with certain personality traits, such as lower self‐directedness and higher shyness, merit extra attention to ascertain that they do not forget their medication and that concerns about possible drug side effects are properly addressed. No association was observed between polypharmacy and medication adherence. However, younger patients and those with lower disability levels were more likely to be non‐adherent, highlighting the need for targeted education on the importance of adherence in less severe cases to ensure favorable long‐term outcomes. Additionally, our findings suggest that non‐adherence is related to anxiety and depression, common psychopathologies in pwMS that shape both personality and behavior. It may therefore be speculated that an appropriate management of these symptoms could support regular medication intake, alongside strategies to simplify the use of medication in everyday life.

## Author Contributions

N.F., J.R., F.H., and U.K.Z. conceptualized the study. P.M., N.F., J.B., K.B., S.E.L., J.M., and B.S. collected the data. M.H. analyzed the data and prepared the figures and tables. P.M. and N.F. have verified the underlying data. P.M. and M.H. interpreted the data and drafted the original manuscript. N.F., B.B., A.M.S., and U.K.Z. provided important intellectual content. U.K.Z. supervised the research. All authors have read and approved the final version of the manuscript.

## Funding

The authors have nothing to report.

## Ethics Statement

Ethical approval for the study was obtained from the ethics committee of the University of Rostock (permit number: A 2019‐0048), with additional approval from the Medical Chamber of Thuringia. The study was conducted following the principles of the Declaration of Helsinki and in accordance with the European data protection regulations.

## Consent

All patients participated on a voluntary basis and provided written informed consent prior to participation.

## Conflicts of Interest

M.H. received speaking fees and travel funds from Bayer HealthCare, Biogen, Merck Healthcare, Novartis, and Teva. N.F. received travel funds for research meetings from Novartis. U.K.Z. received research support, speaking fees, and travel funds from Alexion, Almirall, Bayer HealthCare, Biogen, Bristol Myers Squibb, Janssen, Merck Healthcare, Novartis, Roche, Sanofi Genzyme, and Teva, as well as the European Union, BMBF, BMWi, and DFG. P.M., J.B., B.B., K.B., S.E.L., J.M., B.S., A.M.S., J.R., and F.H. declare no conflicts of interest.

## Supporting information


**Table S1:** Comorbidities of the patients with multiple sclerosis.

**Table S2:** Drug compounds that were used by the patients with multiple sclerosis.

## Data Availability

The raw data supporting the findings of this study are available from the corresponding author upon request.
